# The Expression Level of CB_1_ and CB_2_ Receptors Determines Their Efficacy at Inducing Apoptosis in Astrocytomas

**DOI:** 10.1371/journal.pone.0008702

**Published:** 2010-01-14

**Authors:** Eiron Cudaback, William Marrs, Thomas Moeller, Nephi Stella

**Affiliations:** 1 Department of Pharmacology, University of Washington, Seattle, Washington, United States of America; 2 Graduate Program in Neurobiology and Behavior, University of Washington, Seattle, Washington, United States of America; 3 Department of Neurology, University of Washington, Seattle, Washington, United States of America; 4 Department of Psychiatry and Behavioral Sciences, University of Washington, Seattle, Washington, United States of America; City of Hope National Medical Center, United States of America

## Abstract

**Background:**

Cannabinoids represent unique compounds for treating tumors, including astrocytomas. Whether CB_1_ and CB_2_ receptors mediate this therapeutic effect is unclear.

**Principal Findings:**

We generated astrocytoma subclones that express set levels of CB_1_ and CB_2_, and found that cannabinoids induce apoptosis only in cells expressing low levels of receptors that couple to ERK1/2. In contrast, cannabinoids do not induce apoptosis in cells expressing high levels of receptors because these now also couple to the prosurvival signal AKT. Remarkably, cannabinoids applied at high concentration induce apoptosis in all subclones independently of CB_1_, CB_2_ and AKT, but still through a mechanism involving ERK1/2.

**Significance:**

The high expression level of CB_1_ and CB_2_ receptors commonly found in malignant astrocytomas precludes the use of cannabinoids as therapeutics, unless AKT is concomitantly inhibited, or cannabinoids are applied at concentrations that bypass CB_1_ and CB_2_ receptors, yet still activate ERK1/2.

## Introduction

Cannabinoids produce the majority of their biological effects by activating two receptors: CB_1_ and CB_2_. In healthy brain, CB_1_ is expressed by neurons, astrocytes and neural progenitor cells, whereas CB_2_ is only expressed by a small population of neurons located in the brain stem [1], [2]. Activation of CB_1_ and CB_2_ receptors regulates fundamental physiological processes, including neurotransmission efficacy, astrocytic function, and the fate and proliferation rate of neural progenitor cells [3], [4]. Thus, because these receptors regulate such fundamental physiological processes, extensive effort has been invested toward understanding how cannabinoid compounds activate CB_1_ and CB_2_ receptors, as well as regulate the signal transduction mechanisms they couple to, with the goal of developing novel therapeutics to treat various neuropathologies [5], [6].

One of the most promising therapeutic uses of cannabinoids is linked to their ability to induce apoptosis in tumors, including in astrocytoma subclones grown either *in vitro*, subcutaneously in immunodeficient mice, or intracranially in rats [7], [8]. In fact, these data were so compelling that one clinical trial has already been carried-out, in which patients afflicted with recurrent astrocytomas (grade IV) were treated with cannabinoids stereotactically injected directly into the mass of the malignant tumors [9]. While there are clear results for the use of cannabinoids to treat astrocytomas, the requirement of cannabinoid receptors in mediating this therapeutic effect is still unproven [10]. In fact, evidence suggests that high concentrations of cannabinoids may induce biological effects, including the induction of apoptosis in cells, independently of CB_1_ and CB_2_ receptors [7], [11], [12], [13]. Thus whether CB_1_ and CB_2_ receptors, or yet another cannabinoid-like receptor, mediate the therapeutic effects of cannabinoids toward astrocytomas is still unclear.

Other unanswered questions are: Why does CB_1_ and CB_2_ expression in astrocyomas gradually increase as a function of cancerous transformation? [14], [15] Does this increase in receptor expression modify the coupling of CB_1_ and CB_2_ to signal transduction pathways in astrocytomas? Does this increase in receptor expression modify the efficacy of cannabinoids at inducing apoptosis in astrocytomas? Indeed, while it is known that CB_1_ and CB_2_ couple to ERK1/2, AKT, JNK, p38 and GSK-3β [8], [16], [17], [18], [19], and that both ERK1/2 and AKT mediate the induction of apoptosis by cannabinoids in astrocytomas [8], [11], [18], [20], nothing is known about how changes in CB_1_ and CB_2_ expression will modify the coupling of these receptors to each kinase, and whether the efficacy with which cannabinoids induce apoptosis is also affected. Answering these questions is important for the following reasons. Most of the studies reporting cannabinoid receptors coupling to specific kinases were performed in heterologous expression systems and the expression level of these receptors both surpass those reached by endogenously-expressed receptors and favor promiscuous coupling to effector proteins. One particularly controversial point stems from the ability of cannabinoids to induce apoptosis in astrocytomas when CB_1_ and CB_2_ receptors also couple to the pro-survival kinase AKT. Therefore, it is important to increase our understanding of how changes in the expression level of CB_1_ and CB_2_ will affect the coupling of these receptors to specific kinases, as well as the efficacy of cannabinoids at killing astrocytomas.

Here we sought to address the following two questions: Are the therapeutic effects of cannabinoids toward astrocytomas truly mediated by CB_1_ and CB_2_ receptors? Does the increase in cannabinoid receptor expression known to occur in malignant astrocytomas determine the efficacy of cannabinoids at killing these tumors? To answer these questions, we generated astrocytoma subclones that express low, medium and high levels of either CB_1_ or CB_2_, and studied the ability of the cannabinoid agonist CP-55,940 to regulate the activity of various kinases and to induce apoptosis in these tumor cells.

## Results

### Astrocytoma Subclones Stably Expressing Set Levels of Either CB_1_ or CB_2_ Receptors

To determine whether the expression level of CB_1_ or CB_2_ receptors dictate cannabinoid-mediated responses in astrocytomas, we sought to use heterologous gene expression technology to drive the expression of these receptors to precise levels in an astrocytoma cell line that normally lacks these receptors. Specifically, we found that the murine delayed brain tumor (DBT) cell line expresses neither CB_1_ nor CB_2_ receptors (Table 1). Thus we transfected these cells with IRES constructs containing the gene that encodes either CB_1_ or CB_2_ receptors in tandem with the gene encoding eGFP, and used FACS to select for the cells that had stably incorporated the construct (see Experimental Procedures). Using this approach, we obtained 27 CB_1_-expressing DBT subclones and 49 CB_2_-expressing DBT subclones. We then randomly selected 24 subclones (twelve CB_1_ and twelve CB_2_) and performed qPCR to find out the relative expression level of CB_1_ and CB_2_ receptor mRNA in each single cell subclone (Table S1). Based on these data, we then focused our experiments on three CB_1_ subclones and three CB_2_ subclones that expressed relatively low, medium and high levels of receptor mRNA (Table 1). Please note that all CB_1_-expressing DBT cells were still devoid of CB_2_ receptor mRNA, and *vice versa*. Quantification of CB_1_ and CB_2_ receptor proteins by radioligand binding showed a correlation between the relative level of mRNA encoding each receptor and the corresponding protein expression level as expressed by the B_max_ value (Table 1). Here it is worth noting that the expression level of CB_1_ and CB_2_ receptors in the CB_1_-mid and the CB_2_-mid subclones, respectively, lie well within the range of their levels when endogenously expressed by various cell lines and tissues [21], [22]. Thus, we generated DBT subclones that stably express set levels of either CB_1_ or CB_2_ receptors, with expression levels that lie within the range of what is found for cells endogenously expressing these receptors.

**Table 1:** Expression levels of CB_1_ and CB_2_ receptors in wild-type and selected DBT subclones.

Nomenclature	subclones	qPCR		Radioligand binding	
		CB_1_	CB_2_	B_max_	k_d_
		(cycle threshold)		(pmol/mg)	(nM)
Wild type	WT	*no ct*	*no ct*	*no binding*	-
CB_1_-low	E3	22.4	*no ct*	0.2	0.8
CB_1_-mid	E7	17.7	*no ct*	0.7	0.8
CB_1_-high	E5	14.7	*no ct*	1.3	0.7
CB_2_-low	2G5	*no ct*	24.1	0.3	0.8
CB_2_-mid	2H1	*no ct*	18.0	0.6	0.4
CB_2_-high	1D6	*no ct*	14.0	2.3	0.6

Wild-type DBT cells and DBT subclones stably expressing cannabinoid receptors were expanded in 10 cm dishes, and either their RNA extracted for qPCR analysis of CB_1_ and CB_2_ mRNA levels, or homogenized for radioligand binding analysis of CB_1_ and CB_2_ receptor protein levels. mRNA levels are expressed as *cycle threshold* (ct) value from qPCR performed with Taqman probes. Values represent mean of three independent determinations. Receptor protein level is expressed as *B_max_* values from radioligand binding experiments performed with [^3^H]CP-55,940. *k_d_* values were also determined and show consistent low nanomolar affinities. Values represent mean of 3–5 independent determinations.

### Independent of Expression Levels, CB_1_ and CB_2_ Receptors Similarly Regulate ERK1/2 Signaling

**Figure 1:**
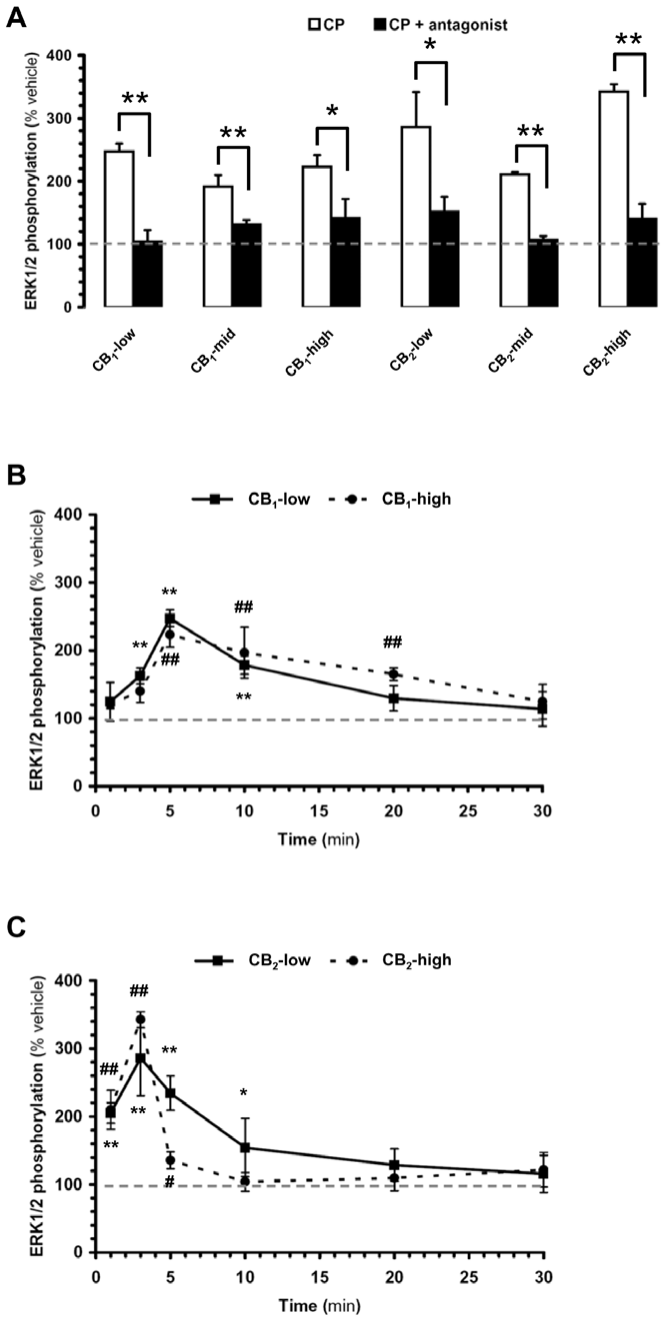
Activation of CB_1_ and CB_2_ receptors increases ERK1/2 phosphorylation in all DBT subclones. DBT subclones stably expressing cannabinoid receptors were expanded in 24-well plates, incubated with CP-55,940 (CP, 1 µM), and ERK1/2 phosphorylation quantified by Western blot analysis. *(a)* CB_1_- and CB_2_-expressing subclones were incubated with CP for 5 and 3 min, respectively (white bars). When testing the effect of antagonists, cells were pretreated with SR141716A (5 µM) and SR144528 (3 µM) added 10 min before CP (black bars). *(b,c)* Kinetics of CP-induced increase in ERK1/2 phosphorylation in CB_1_-low, CB_1_-high, CB_2_-low and CB_2_-high subclones. Data = mean±s.e.m. of 6–8 independent experiments expressed as % of vehicle (*i.e.* level of ERK1/2 phosphorylation when treated with vehicle, *i.e.* 0.1% DMSO. Note that basal ERK1/2 phosphorylation did not vary significantly over time (Figure S1a). In *(a)*, (*) = p<0.05 and (**) = p<0.01 significantly different from the response in the presence of inhibitor, ANOVA followed by Bonferroni's post-test. In *(b,c)*, (*) and (#) = p<0.05, and (**) and (##) = p<0.01 significantly different from vehicle at corresponding time point, ANOVA followed by Bonferroni's post-test.

Because previous studies performed on astrocytoma cell lines have implicated ERK1/2 signaling in the therapeutic actions of cannabinoids [23], we sought to determine whether different expression levels of CB receptors would affect their coupling to this signal transduction pathway. To do so, we treated each subclone with CP-55,940 (1 µM, maximally efficacious concentration [17], [24]) and measured ERK1/2 phosphorylation by Western blot analysis. We found that CP-55,940 induced a significant increase in ERK1/2 phosphorylation above vehicle-treated controls in all six subclones (Figure 1a). All these responses were receptor-mediated since they were significantly attenuated by either the CB_1_ antagonist SR141716A, or the CB_2_ antagonist SR144528, respectively (Figure 1a). Furthermore, CP-55,940 did not affect ERK1/2 phosphorylation in wild-type DBT cells (Figure S1a). The magnitude and kinetics of CP-55,940-induced ERK1/2 phosphorylation mediated in each subclone were remarkably similar despite a 10-fold difference in receptor expression level between the CB_1_-low and the CB_1_-high subclones, and between the CB_2_-low and the CB_2_-high subclones (Figure 1b,c). The only differences that we noted between CB_1_- and CB_2_-mediated regulation of ERK1/2 phosphorylation were that CP-55,940 induced a slightly slower and sustained increase in ERK1/2 phosphorylation in CB_1_-expressing subclones (peak at 5 min returning to basal by 20–30 min) compared to CB_2_-expressing subclones (peak at 3 min returning to basal by 10–20 min) (Figure 1b,c). These results show that CB_1_ and CB_2_ receptors heterologously expressed in DBT cells are functional for they regulate ERK1/2 phosphorylation. They also show that the efficacy of this regulation is largely independent of receptor expression levels and receptor subtype.

### The Expression Level of CB_1_ and CB_2_ Receptors Dictates Their Ability to Regulate AKT, JNK and p38 Signaling

CB_1_ and CB_2_ receptors have been shown to regulate a plethora of kinases, including AKT, JNK, p38 and GSK-3β [8], [16], [17], [18], [19]. Thus we sought to assess if CB_1_ and CB_2_ heterologously expressed in DBT cells also regulate these four kinases. To do so, we treated all DBT subclones with CP-55,940 and assessed the phosphorylation state of these kinases using a Luminex multiplexed immunoassay system.

**Figure 2:**
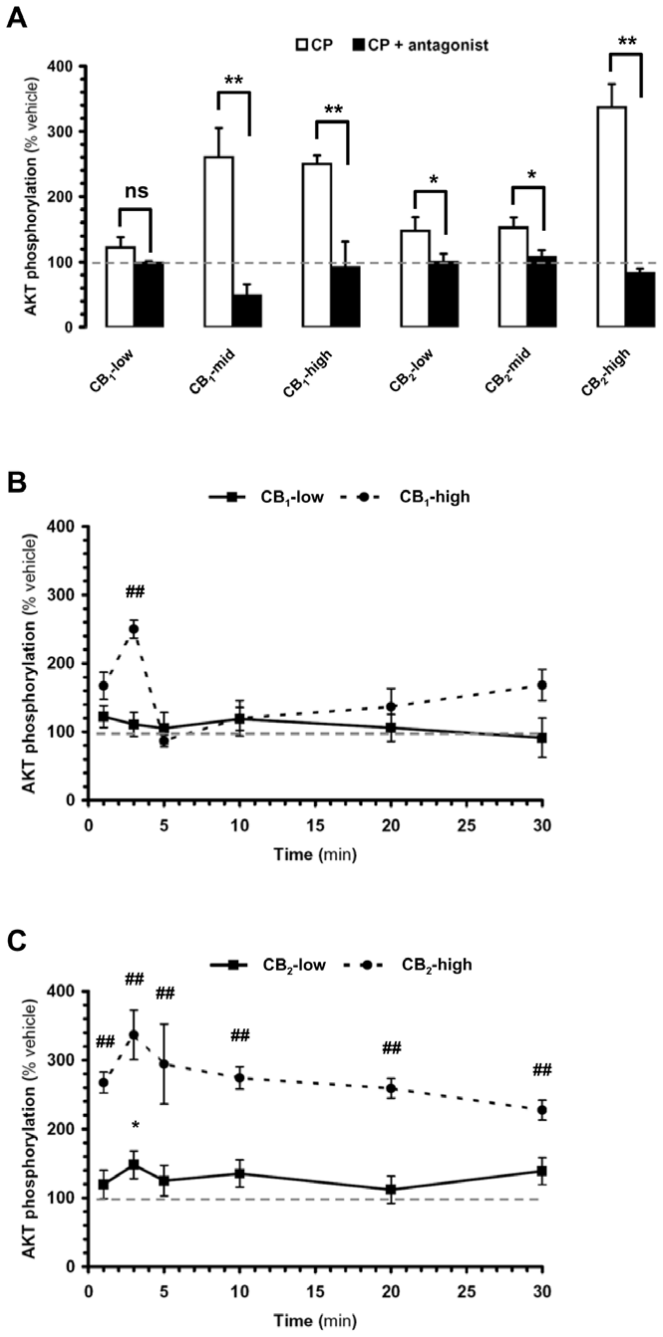
Activation of CB_1_ and CB_2_ receptors differentially increases AKT phosphorylation in DBT subclones. DBT subclones stably expressing cannabinoid receptors were expanded in 24-well plates, incubated with CP-55,940 (CP, 1 µM), and AKT phosphorylation quantified by Luminex multiplex immunoassay. *(a)* CB_1_- and CB_2_-expressing subclones were incubated with CP for 5 and 3 min, respectively (white bars). When testing the effect of antagonists, cells were pretreated with SR141716A (5 µM) and SR144528 (3 µM) added 10 min before CP (black bars). *(b,c)* Kinetics of CP-induced increase in AKT phosphorylation in CB_1_-low, CB_1_-high, CB_2_-low and CB_2_-high subclones. Data = mean±s.e.m. of 6 to 8 independent experiments expressed as % of vehicle (*i.e.* level of AKT phosphorylation when treated with vehicle, *i.e.* 0.1% DMSO. Please note that basal AKT phosphorylation did not vary significantly over time (Figure S1b). In *(a)*, (*) = p<0.05 significantly different from the response in the presence of inhibitor, ANOVA followed by Bonferroni's post-test. In *(b,c)*, (*) and (#) = p<0.05, and (**) and (##) = p<0.01 significantly different from vehicle at corresponding time point, ANOVA followed by Bonferroni's post-test. Non-significant (ns).

With regard to AKT, three points are noteworthy. First, the CP-55,940-induced increase in AKT phosphorylation correlated with receptor expression levels (Figure 2a). Specifically, CP-55,940 did not significantly increase AKT phosphorylation in the CB_1_-low subclone, which expresses the lowest level of receptor of all six subclones that we have selected; it induced a small, but significant increase in AKT phosphorylation in CB_2_-low and CB_2_-mid, which express slightly more receptors than the CB_1_-low subclone; and it induced the most pronounced increase in AKT phosphorylation in CB_1_-mid, CB_1_-high and CB_2_-high subclones (see Table 1 for B_max_ values). Second, all responses were mediated by cannabinoid receptors since they were antagonized by either SR141716A or SR144528, respectively, and CP-55,940 did not increase AKT in wild type DBT cells (Figures 2a and S1b). Third, there was a clear difference in the kinetics of AKT activation linked to either CB_1_ or CB_2_ receptors. Specifically, while the response obtained in the CB_1_-high subclone peaked at 3 min and returned to the basal level at 5 min, the response measured in the CB_2_-high clone remained above 250% of basal during the entire 30 min of the incubation period (Figure 2b,c).

With regard to JNK and p38, CP-55,940 induced a small (150–170% of basal) but significant increase in the phosphorylation state of these kinases only in the CB_2_-high clone that also lasted throughout the 30 min incubation period (Figure S2a,b). With regard to GSK-3β, CP-55,940 did not significantly affect the phosphorylation state of this kinase in any of the subclones throughout the 30 min incubation time period (data not shown).

Taken together, these results suggest that the different expression levels of CB_1_ and CB_2_ receptors determine the efficacy with which cannabinoids regulate AKT, JNK and p38 signaling. They also suggest that CB_1_ and CB_2_ receptors differentially couple to these kinases.

### Only Activation of CB_1_ Receptors Expressed at Low Levels in DBT Cells Leads to Apoptosis: Involvement of ERK1/2

**Figure 3:**
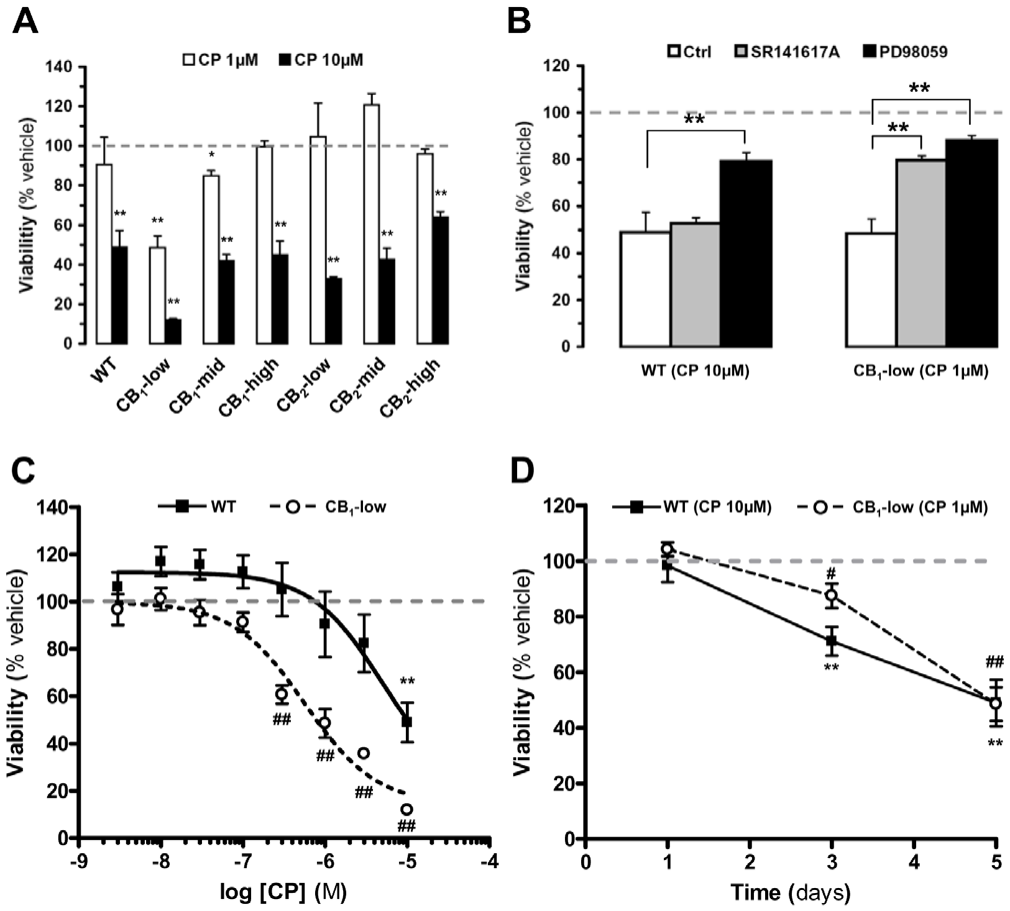
CP-55,940 kills DBT cells via cannabinoid receptor-dependent and -independent mechanisms. Wild-type (wt) DBT cells and DBT subclones stably expressing cannabinoid receptors were expanded in 24-well plates, incubated with CP-55,940 (CP) at either 1 µM (receptor mediated) or 10 µM (receptor independent), and cell viability was assessed by quantifying WST-1 conversion. *(a)* Five days after treatment, only CB_1_-low DBT cells showed significant sensitivity to CP at 1 µM, whereas all subclones were sensitive to CP at 10 µM. Data represent means±s.e.m. of 5–9 independent experiments in triplicate. *(b)* Only the pretreatment of CB_1_-low DBT cells with SR141617A (5 µM) prevented the CP-induced killing, whereas pretreatment with PD 98059 (PD, 10 µM) significantly reduced both the CP 1 µM and CP 10 µM induced toxicity in CB_1_-low and wild-type, respectively. Data represent means±s.e.m. of 3–5 independent experiments in triplicate. *(c)* Dose-response and *(d)* kinetics of CP-induced killing of wt and CB_1_-low DBT cells. Data represent means±s.e.m. of 3–9 independent experiments in triplicate. All results are expressed as % of WST-1 measurements in corresponding cells treated with vehicle (0.1% DMSO) for the indicated time point. In *(a)*, (*) = p<0.05 and (**) = p<0.01 significantly different from the viability in the presence of vehicle only, ANOVA followed by Bonferroni's post-test. In *(b)*, (**) = p<0.01 significantly different the viability after treatment with either 1 or 10 µM CP, ANOVA followed by Bonferroni's post-test. In *(c)*, (**) and (##) = p<0.01 significantly different from the viability in the presence of vehicle only, ANOVA followed by Bonferroni's post-test. In *(d)*, (#) = p<0.05, and (**) and (##) = p<0.01 significantly different from the viability in the presence of vehicle only at corresponding time point, ANOVA followed by Bonferroni's post-test.

Several studies suggest that activation of CB_1_ or CB_2_ is sufficient to induce apoptosis in astrocytomas [8], [14], whereas other studies suggest that only high concentrations of cannabinoids kill astrocytomas and furthermore, independently of cannabinoid receptor activation [7], [11], [12], [13]. We directly tested these possibilities by treating the DBT subclones, as well as wild-type DBT cells, with CP-55,940 at either 1 µM (receptor-mediated) or 10 µM (receptor-independent). To assess for cell viability, we used WST-1, an indicator of mitochondrial activity. We found that 1 µM CP-55,940 induced ∼50% cell death in the CB_1_-low DBT subclone, whereas 10 µM CP-55,940 induced 35–90% cell death in wild type DBT cells and all six subclones (Figure 3a). This result suggests that only the activation of CB_1_ receptors expressed at low levels in DBT cells leads to significant receptor-dependent killing of these cells. Two additional sets of results support this conclusion. First, SR141617A blocked the 1 µM CP-55,940-induced killing of the CB_1_-low DBT cells, whereas this antagonist did not affect the 10 µM CP-55,940-induced cell death of wild type DBT cells (Figure 3b). Second, CP-55,940 killed CB_1_-low DBT cells with an EC_50_ of 0.56 µM, whereas this compound induced the cell death of wild type DBT cells with an EC_50_ of 5 µM (Figure 3c). Interestingly, when comparing the effect of 1 µM CP-55,940 on CB_1_-low DBT cells to the effect of 10 µM CP-55,940 on wild-type DBT, we found that the 10 µM CP-55,940-induced killing of wild-type DBT (receptor-independent) is more rapid than the CB_1_-mediated cell death (Figure 3d).

**Figure 4:**
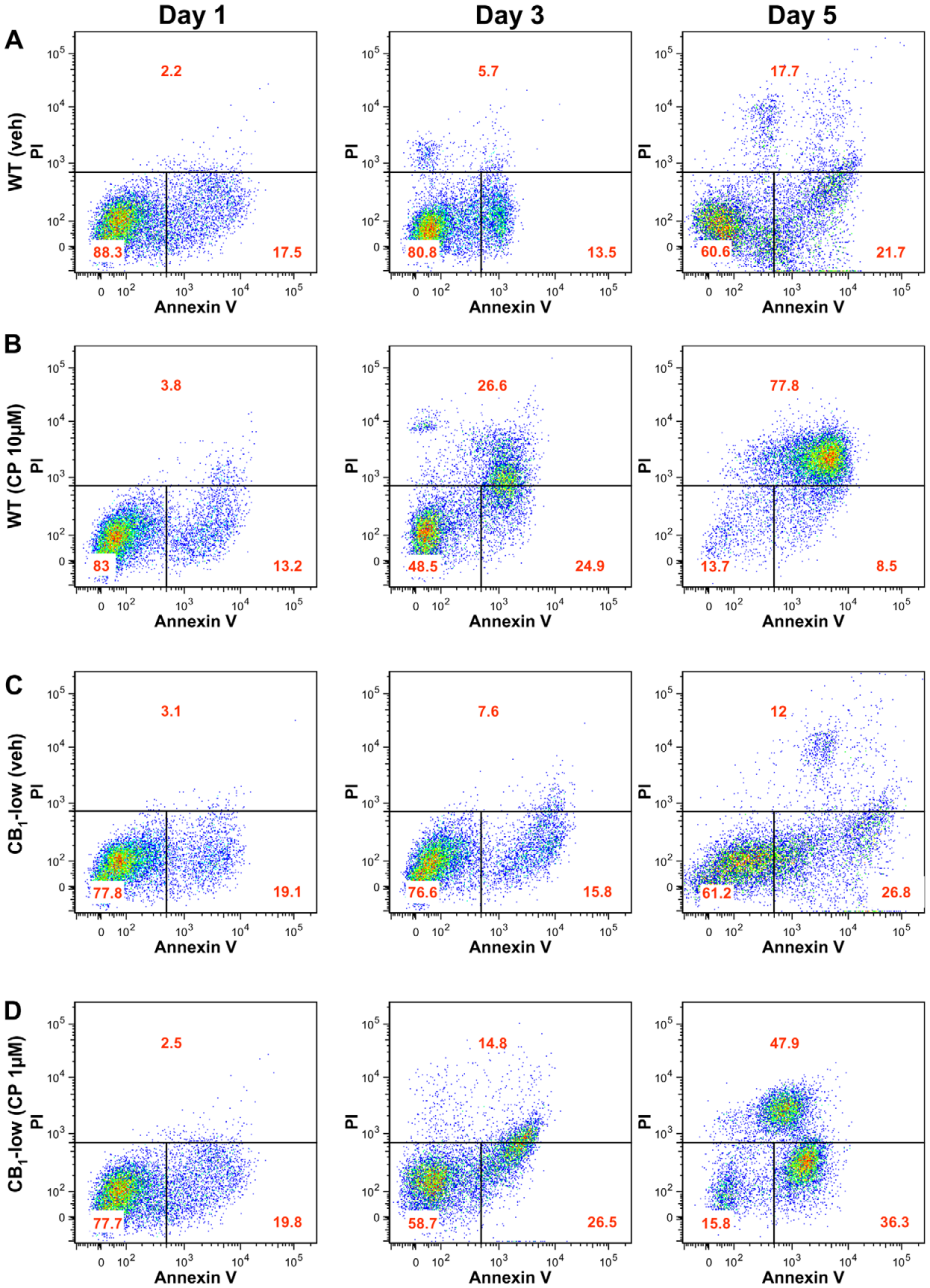
Time course of the apoptotic response induced by CP-55,940 in wild-type and CB_1_-low DBT cells. *(a,b)* Wild-type (wt) DBT cells and *(c,d)* CB_1_-low DBT cells were expanded in 6-well plates, incubated with CP-55,940 (CP) at 10 and 1 µM, respectively, and apoptosis assessed by quantifying propidium iodine (PI) and annexin V fluorescence by FACS after 1, 3, and 5 days. Shown are representative results, with values within each quadrant = mean of 3–5 independent experiments.

We then determined whether the CB_1_-dependent and the cannabinoid receptor-independent killing of DBT cells were apoptotic in nature. Specifically, we treated CB_1_-low DBT cells with 1 µM CP-55,940 and wild type DBT cells with 10 µM CP-55,940, and assessed for propidium iodide and annexin V staining (to assess for cell permeability and apoptosis, respectively). Figure 4 shows that after five days, cell death by apoptosis was induced in 78% of the wild-type DBT cells treated with 10 µM CP-55,940 compared to 48% of the CB_1_-low DBT cells treated with 1 µM CP-55,940. Furthermore, in agreement with the data we have obtained with WST-1, 10 µM CP-55,940 killed more wild-type DBT cells by apoptosis (27%) 3 days after treatment compared to CB_1_-low DBT cells treated with 1 µM CP-55,940 (15%) (Figure 4). Together, these data show that both the CB_1_-dependent and the cannabinoid receptor-independent killing of DBT cells are due to apoptosis, but that these processes occur with slightly different time courses.

Because we found that 1 µM CP-55,940 increases ERK1/2 signaling in CB_1_-low DBT cells without affecting the AKT, JNK, p38 and GSKβ signaling pathways, and one study suggested that ERK1/2 mediates the apoptotic properties of cannabinoids on astrocytomas [8], we then tested if inhibiting ERK signaling would affect the CB_1_-mediated and the cannabinoid receptor-independent killing of DBT cells. Thus, we pretreated CB_1_-low and wild-type DBT cells with the ERK kinase kinase inhibitor PD98059 before applying either 1 or 10 µM CP-55,940. We found that both responses were partially prevented by this inhibitor (Figure 3b). Thus both the CB_1_-dependent and the cannnabinoid receptor-independent induced apoptosis of DBT cells rely on ERK signaling.

Together, these results show that cannabinoid concentrations known to activate CB_1_ and CB_2_ receptors (here 1 µM) induce apoptosis in astrocytomas only when these cells express low levels of CB_1_ receptors, and that this response is mediated through ERK signaling. Conversely, high concentrations of cannabinoids known to bypass cannabinoid receptors (here 10 µM) induce apoptosis in all DBT subclones, a mechanism that also depends on ERK signaling.

### CB_1_- and CB_2_-Induced Apoptosis of DBT Cells Is Uncovered When AKT Is Inhibited

**Figure 5:**
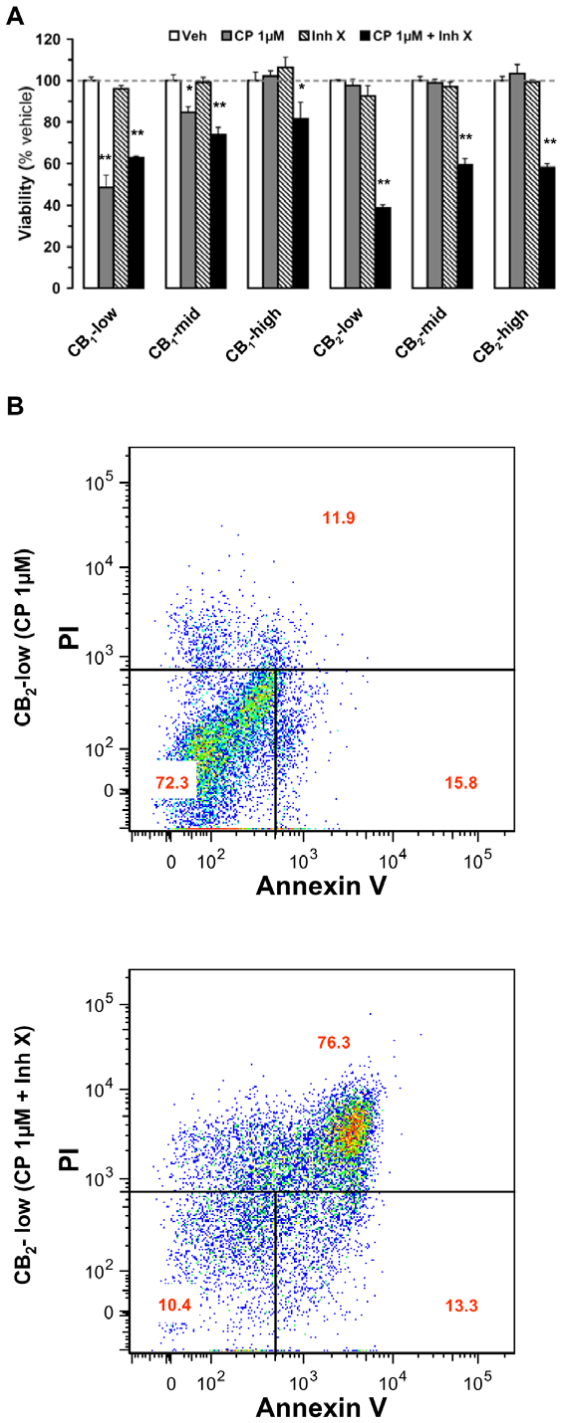
Inhibition of AKT signaling confers sensitivity of cannabinoid receptor-expressing DBT cells to cannabinoid-dependent cytotoxicity. *(a)* Wild-type (wt) DBT cells and DBT subclones stably expressing cannabinoid receptors were expanded in 24-well plates, incubated with CP-55,940 (CP) at 1 µM (receptor-mediated) without or with Inhibitor X (5 µM added 10 min before CP), and cell viability was assessed after 5 days by quantifying WST-1 conversion. Data represent means±s.e.m. of 3–9 independent experiments in triplicate. (*) = p<0.05 and (**) = p<0.01 significantly different from the viability in the presence of vehicle only, ANOVA followed by Bonferroni's post-test. *(b)* wt and CB_2_-low DBT cells were expanded in 6-well plates, incubated with CP at 1 µM (receptor-mediated) without or with Inhibitor X (5 µM added 10 min before CP), and the apoptotic response assessed after 5 days by propidium iodine (PI) and annexin V staining by FACS after 1, 3, and 5 days. Shown are representative results, with values within each quadrant = mean of 3–5 independent experiments.

Because increased AKT signaling promotes cell survival [25], we reasoned that activation of this pathway by CB_1_ and CB_2_ receptors expressed at mid and high levels might explain why 1 µM CP-55,940 was unable to induce apoptosis in DBT subclones. To test this hypothesis, we treated the six subclones of DBT cells with 1 µM CP-55,940 in combination with Inhibitor X, a cell-permeable inhibitor of AKT [26]. Using the WST-1 assay, we found that while this inhibitor had no effect by itself on cell viability, it uncovered the ability of 1 µM CP-55,940 to kill all the DBT subclones that were normally resistant, with the most pronounced response occurring in CB_2_-low DBT cells (Figure 5a). This latter response was due to apoptosis, since 76% of the cells stained positively for annexin V when measured five days after co-application of 1 µM CP-55,940 and Inhibitor X (Figure 5b). These results suggest that the coupling of CB_1_ and CB_2_ receptors to the AKT pathway (when these receptors are expressed at mid and high levels) eliminates the ability of cannabinoids to induce apoptosis in DBT cells.

## Discussion

While many laboratories have reported that cannabinoids induce apoptosis in various tumor subtypes, including astrocytomas, the requirement for CB_1_ and CB_2_ in mediating this therapeutic effect has not yet been demonstrated. This point is especially relevant when considering that many of these studies used high concentrations of cannabinoids known to bypass CB_1_ and CB_2_ receptor activation. Here we demonstrate that cannabinoid receptors and ERK1/2 indeed mediate this therapeutic effect, but only when these compounds are applied at submicromolar concentrations and the expression level of these receptors remains low. Conversely, high concentrations of cannabinoids kill all astrocytoma subclones independently of CB_1_, CB_2_ and AKT, yet still through a mechanisms involving ERK1/2. We also show that increased expression of CB_1_ and CB_2_ receptor allows for their coupling to additional kinases, especially AKT, the result of which eliminates the ability of cannabinoids to induce apoptosis even though these receptors still couple to ERK1/2.

One of the most unexpected and interesting findings of our study is that the coupling of CB_1_ and CB_2_ receptors to AKT, JNK and p38 is dictated by receptor expression levels, whereas their coupling to ERK1/2 is not. What may be the molecular mechanism underlying a gradual coupling to AKT, JNK and p38, while preserving a steady coupling to ERK1/2? Reports on the molecular mechanisms linking cannabinoid receptors to various kinases suggest that these receptors activate AKT through G-proteins, while activating ERK1/2 through EGF receptor transactivation [27]. Based on these reports, a parsimonious interpretation of our results would be that the rate limiting step controlling the efficacy of cannabinoids at stimulating AKT lies in the cannabinoid receptors themselves, whereas the rate limiting step controlling the efficacy of cannabinoids at stimulating ERK lies in EGF receptors, the expression of which is likely to remain constant in the DBT subclones that we generated. Our results may have broader implications when considering the therapeutic efficacy of cannabinoids toward other neuropathologies. For example, epileptic seizures and cerebral ischemia lead to a 3–4 fold increase in the expression of neuronal CB_1_ receptors, a response thought to constitute a protective mechanism [28], [29], whereas Huntington disease leads to a 50–70% decrease in the expression of neuronal CB_1_ receptors, a decrease thought to participate in the pathogenesis of this neurodegenerative disease [30], [31]. Based on the results that we report here, one would predict that up- or down-regulation of neuronal CB_1_ receptor expression should not affect the efficacy of cannabinoids at stimulating ERK signaling, whereas it might dictate their ability to modulate the activity of the prosurvival kinase AKT. Thus our study is the first to suggest that changes in cannabinoid receptor expression levels will affect only certain types of signal transductions pathways, several of which are clearly involved in the control of cell survival and death. This result has implications when considering the efficacy of cannabinoids at treating astrocytomas of increasing malignancy, but also when considering other neuropathologies associated with changes in cannabinoid receptor expression.

An additional noteworthy result was the differential coupling of CB_1_ and CB_2_ receptors to AKT. Indeed, cells expressing similar ranges of CB_1_ and CB_2_ receptors (CB_1_-mid to CB_1_-high being between 0.7 and 1.3 pmol/mg, and CB_2_-mid to CB_2_-high being between 0.6 and 2.3 pmol/mg) exhibited a clearly different coupling to this prosurvival kinase. How might the same agonist acting at CB_1_ and CB_2_ differentially stimulate this kinase? CB_1_ and CB_2_ exhibit significant disparities in their amino acid sequence, particularly in their intracellular domains which interact with G proteins and effector proteins. Indeed, upon their molecular identification, it was evident that while both receptors exhibit similar pharmacological profiles, they only exhibited an overall 44% amino acid identity [32]. We preformed a sequence alignment between mouse CB_1_ and mouse CB_2_, and highlighted in red the sequence motifs located in the intracellular loops that may account for the differential interactions with G proteins and found that these regions exhibit a much diminished 29% similarity (Figure S3). In agreement with this reduced amino acid similarity in intracellular sequences, a recent study showed that the binding of the same agonist to either CB_1_ or CB_2_ receptors leads to differential activation of G-proteins [33]. Another possible explanation of how the same agonist engaging either CB_1_ or CB_2_ receptors might differentially activate a signal transduction mechanism is based on studies that were obtained with “ligand-assisted protein structure” (LAPS) analysis [34], [35]. Here the positioning of the tricyclic ring of a specific cannabinoid agonist, AM841 (a close analogue of CP-55,940), within the binding pockets of either CB_1_ or CB_2_ receptors has been shown to vary considerably, from being parallel to the transmembrane helices in CB_1_ to being perpendicular to the transmembrane helices in CB_2_ [34], [35]. Since agonists engaging the binding pocket of a GPCR will favor a specific R* activation conformation, the same agonist differentially engaging the binding pocket of either CB_1_ or CB_2_ receptors will likely lead to distinct R* conformations for these receptors, and thus differential G protein coupling. Hence, both sequence disparities in the intracellular loops and the precise positioning of a cannabinoid ligand within the binding pocket of CB_1_ and CB_2_ receptors could account for the differential activation of a specific kinase.

When considering cannabinoids to treat astrocytomas, many have attempted to isolate the non-psychotropic therapeutic effects ascribed to CB_2_ receptor activation, while eliminating the psychotropic and addictive properties ascribed to CB_1_ receptor activation [14]. However, since cannabinoids have outstanding LD_50_ values (mainly because cannabinoid receptors are barely expressed in brains regions that control vegetative functions) [36], the treatment of tumors with high concentrations of cannabinoids should not be overlooked. In fact, stereotaxic injection of high concentrations of cannabinoids will eradicate a significant portion of C6 astrocytomas inoculated into rodent brains without affecting healthy surrounding tissue or inducing overt adverse effects [8]. Since stereotaxic injection of chemotherapeutic compounds directly into human brain tumor masses constitutes a routine approach for neurosurgeons, high concentrations of cannabinoids can easily be delivered by this technique [9]. There are two additional advantages to delivering high concentrations of cannabinoids directly into the tumor mass. Malignant transformation of astrocytomas is associated with an increase in cannabinoid receptor expression [14], [15] and we show here that this increase in expression precludes the use of low concentrations of cannabinoids known to activate these receptors. Conversely, local injection of high concentrations of cannabinoids will induce apoptosis in all astrocytoma subclones (independently of CB_1_ and CB_2_ receptor expression), which constitutes an asset when considering the phenotypic heterogeneity that astrocytomas adopt during malignant transformation [37], [38]. Thus, our results suggest that high concentrations of cannabinoids constitute the preferred regimen for neurosurgeons to use when treating malignant astrocytomas with this class of compounds.

One outstanding question that is raised by our study is: what constitutes the molecular target that is engaged by high concentrations of cannabinoids, a target that also leads to ERK activation and induction of apoptosis in astrocytomas? One possibility is another GPCR, several of which have recently been shown to be engaged by high concentrations of cannabinoids, including CP-55,940 [39]. One obvious candidate is GPR55 [40], although this receptor can be ruled out in the context of our studies since we found no evidence for the presence of GPR55 mRNA in DBT cells (as assessed by RT-PCR and qPCR, data not shown). Thus, while the molecular identification of this target remains to be determined, its identification will most certainly raise a lot of interest for research aimed at developing novel therapeutic approaches to treat astrocytomas and other tumors.

In conclusion, our study provides the first evidence for the differential activation of AKT, p38 and JNK by a highly efficacious cannabinoid agonist, CP-55,940, engaging either CB_1_ or CB_2_ receptors expressed at different levels. This shift in coupling precludes the use of cannabinoids at submicromolar concentrations as pro-apoptotic therapeutics, unless AKT is concomitantly inhibited. Our results also suggest that high concentrations of cannabinoids are preferable for efficacious treatment of malignant astrocytomas, because these concentrations bypass CB_1_ and CB_2_ receptor activation and induce apoptosis in all astrocytoma cell subpopulations.

## Materials and Methods

### Drugs

Tritium-labeled and unlabeled CP 55,940 ((−)-*cis*-3-[2-hydroxy-4-(1,1-dimethylheptyl)phenyl]-*trans*-4-(3-hydroxypropyl) cyclohexanol), SR141716A (5- (4-chlorophenyl)-1-(2,4-dichlorophenyl)-4-methyl-*N*-(1-piperidyl)-1*H*-pyrazole-3-carboxamide), and SR144528 (5- (4-chloro-3- methyl- phenyl)-1- [(4- methylphenyl)methyl]- N-(1,3,3- trimethylnorbornan- 2- yl)- pyrazole- 3- carboxamide) were provided by the National Institute of Drug Abuse Drug Supply Program (RTI, Research Triangle Park, NC). PD 98059 and Inhibitor X were from CalBiochem (San Diego, CA).

### Generation of DBT Subclones Expressing mCB_1_ and mCB_2_

mCB_1_ and mCB_2_ were PCR-cloned from an existing construct using the following primers, respectively: forward, 5′-gcgaattcatgaagtcgatcttagacggccttgc-3′ and reverse, 5′-gcggatcctcacagagcctcggcagacgtgtctg-3′; forward, 5′-gcgaattcatggagggatgccgggagacagaagtg-3′ and reverse, 5′-gcggatcctaggtggttttcacatcagcctctg-3′. Please note that these receptors are not linked to any molecular tag, because such additions often affect the coupling of GCPRs to their signal transduction pathways. Amplicons were digested with EcoR I and BamH I, and ligated into the corresponding multiple cloning site of the pIRES2-EGFP vector (BD Biosciences Clontech, Mountain View, CA). The murine delayed brain tumor (DBT) cell line, which was originally developed using the Schmidt-Ruppin strain of Rous sarcoma virus inoculated into an adult CDF1 mouse brain [41], was expanded at 37°C with 5% CO_2_ in 10 cm Falcon dishes (BD Biosciences, San Jose, CA) containing 10 ml of culture media: DMEM+GlutaMAX™-I (Gibco, Carlsbad, CA) supplemented with fetal bovine serum (FBS, 10%), HEPES (10 mM) and penicillin/streptomycin (100 units/ml and 100 µg/ml, respectively). Once cells reached ∼70% confluence, they were transfected with either pIRES2-EGFP-mCB_1_ or pIRES2-EGFP-mCB_2_ using SuperFect (QIAGEN, Valencia, CA) according to the manufacturer's instructions. Three days after transfection, the resulting positive cells (∼5% of fluorescent green cells) were isolated using fluorescence assisted cell sorting (FACS, BD FACS Vantage SE) (Figure S4). These enriched cells were put back into culture for an additional 7 days of selection and expanded in media containing G418 (10 ml *per* 10 cm culture dishes). Cells that survived were isolated again by FACS, and similarly reselected and expanded for three additional rounds to ensure for the stability of each clone. In the final round, we used FACS to isolate single eGFP-positive cells and seeded them into Costar 96-well plates (0.2 ml *per* well, changing the media every 3–5 days). Three to six weeks later, all single-cell subclones that had expanded were harvested. Please note that both the fluorescence emitted by these single-cell subclones and the receptor expression levels were stable for at least 20 passages, showing that the shuttled plasmid had been stably incorporated in these cells.

### qPCR and Radioligand Binding

Cells (10^6^) were expanded in 10 cm dishes using 10 ml culture media. Once they reached ∼70% confluence, they were rinsed with PBS and kept for an additional 24 hrs in serum-deprived media, which is composed of DMEM+GlutaMAX™-I supplemented with fatty acid free bovine serum albumin (0.1%, BSA, Sigma-Aldrich, St. Louis, MO) and insulin-transferin-sodium selenite liquid media (1×, ITS, Sigma-Aldrich). For qPCR, media was removed and cells washed once with 10 ml PBS, then cells were scraped and total RNA isolated using RNeasy extraction kit (QIAGEN) according to the manufacturer's instructions. The following primer and TaqMan probe sequences were generated using Primer Express (Perkin-Elmer Applied Biosystems, Foster City, CA): mCB_1_ forward, 5′-cacaagcacgccaataacaca-3′, reverse, 5′-acagtgctcttgatgcagctttc-3′, probe, 5′-(gccagcatgcacagggccgcg)-3′; mCB_2_ forward, 5′-gaacatggccgtgctctatattatc-3′, reverse, 5′-gaacaggtacgagggctttctg-3′, probe, 5′-(ctgtcctcccggcggctccg)-3′. Total RNA from each sample (2.5 ng) was reverse transcribed in the same 10 µl quantitative PCR reaction mix using the Brilliant Single-Step Quantitative RT-PCR Core Reagent Kit (Stratagene, La Jolla, CA). Combined reactions were carried out on a Stratagene Mx3000P Real-Time Detection System using the following protocol: 30 min single-strand synthesis reaction at 45°C; 40-cycle, two-step PCR amplification (15 sec at 95°C followed by 1 min at 56°C). Duplicate reactions with and without reverse transcriptase were run for each sample. For radioligand binding, media was removed and cells washed once with 10 ml PBS. Cells were scraped in 1 ml buffer (50 mM Tris-HCl, 3 mM MgCl_2_, 1 mM EDTA, pH 7.4) and pulse homogenized 10 sec using PRO200 tissue homogenizer (PRO Scientific, Oxford, CT). Crude membrane preparations were generated by centrifugation of cell homogenates for 20 min at >10,000×*g*. Cell pellets were resuspended in buffer containing BSA (0.1%, fatty acid free) and 50 µg protein added to siliconized glass tubes for assay. Concentrations of [^3^H]CP-55,940 between 0.1 and 10 nM were used for saturation experiments, and 1 µM CP-55,940 was used to determine specific binding. After 1 hr incubation at 30°C, reactions were stopped by rapid filtration over GF/B glass fiber filters on a 24-well M-24T Brandel cell harvester (Brandel, Gaithersburg, MD) and washed 3 times with ice-cold binding buffer containing BSA. Radioactivity on filters was measured using scintillation counter.

### Quantifying the Phosphorylation of ERK1/2, AKT, JNK, p38 and GSKβ

Cells were expanded in Costar 24-well plates (10^5^ cells *per* well) in culture media (0.5 ml *per* well). Once they reached ∼70% confluence, they were rinsed with PBS and kept for an additional 24 hrs in serum-deprived media, at which time point drugs or vehicle (DMSO, 0.1%) prepared in 50 µl serum-deprived media were directly added to each well for the corresponding number of minutes. We chose to directly administer the drugs to cell culture media instead of replacing the media to avoid stress-induced increases in basal kinase phosphorylation (Figure S1). To quantify Erk phosphorylation, reactions were stopped by removing the media and adding NuPAGE® LDS sample buffer (300 µl, Invitrogen, Carlsbad, CA) supplemented with 2-mercaptoethanol (1%). An aliquot of each sample (20 µl) was loaded onto NuPAGE® 4–12% Bis-Tris pre-cast gels (Inivtrogen) and electrophoresed according to the manufacturer's instructions. Gels were electrophoretically transferred to P-nitrocellulose membrane (Millipore, Billerica, MA) and blocked in LiCOR blocking buffer (LiCOR, Lincoln, NE) for 1 hr at 25°C. Membranes were then incubated in blocking buffer containing rabbit anti-phospho-p44/42 antibody (1∶1000, Cell Signaling Technology, Inc., Danvers, MA) for 1 hr at 25°C. Membranes were washed 3 times with Tris (50 mM) buffered saline containing 0.1% Tween-20 (TBS-T). Membranes were then incubated in blocking buffer containing donkey anti-rabbit antibody conjugated to IRDye 800 (1∶7500, Rockland Immunochemicals, Inc., Gilbertsville, PA) for 1 hr at 25°C. Membranes were washed 3 times with TBS-T. The fluorescence intensity of each band was quantified using the LiCOR Odyssey. To quantify AKT, JNK, p38 and GSKβ phosphorylation, reactions were stopped by addition of NP40 cell lysis buffer (100 µl, Invitrogen). Substrate phosphorylation was quantified using a Biosource Multiplex Bead Immunoassay kit (Invitrogen) according to the manufacturer's instructions and using a Luminex 100™ instrument (Luminex, Austin, TX). Note that the basal phosphorylation state of these kinases in each subclone did not change significantly when the cells were incubated over the 30 min time-period of the incubation with vehicle only (Figure S1).

### Quantification of Cell Viability and Apoptosis by WST-1, Propidium Iodine (PI) and Annexin V

Cells were expanded in 24-well plates (10^5^ cells *per* well) in culture media (0.5 ml *per* well). Once they reached ∼70% confluence, they were rinsed with PBS and kept for an additional 24 hrs in serum-deprived media, at which time drugs or vehicle (DMSO, 0.1%) prepared in 50 µl serum-deprived media were directly added to each well. After 1, 3, or 5 days, cell viability was assessed using the Cell Proliferation Reagent WST-1 (Roche, Indianapolis, IN). Briefly, WST-1 reagent (50 µl) was added to each well for 4 hrs at 37°C with 5% CO_2_, and then WST-1 products were read at 450 nm using Packard SpectraCount™. Apoptosis was assessed using PI and annexin V (Pacific Blue™, Invitrogen). Briefly, cells were detached using trypsin, rinsed with 25°C PBS and resuspended to a density of 10^6^ cells *per* ml of annexin-binding buffer (10 mM HEPES, 140 mM NaCl, 2.5 mM CaCl_2_, pH 7.4). PI (100 ng) and annexin V were added to 100 µl of cell suspension and incubated at 25°C for 30 min. Additional annexin-binding buffer was added (400 µl) and samples were sorted (10,000 events) on a LSR II flow cytometer (BD Biosciences, San Jose, CA) and the data analyzed using FlowJo (Tree Star, Inc., Ashland, OR).

### Data Analysis

Statistics, B_max_, k_d_, and EC_50_ values were calculated using Prism.

## Supporting Information

Figure S1 Kinase activity in wild-type.
(0.45 MB TIF)

Figure S2 p38 and jnk activity.
(0.49 MB TIF)

Figure S3 Sequence alignement between mouse CB_1_ and mouse CB_2_.
(0.77 MB TIF)

Figure S4 Scheme outlining the development of stable clones.
(0.48 MB TIF)

Table S1 qPCR identification of cannabinoid receptor stable subclones.
(0.04 MB DOC)
