# Supplementary figures and images for: The Expression Level of CB1 and CB2 Receptors Determines Their Efficacy at Inducing Apoptosis in Astrocytomas
Source: PLoS One. 2010 Jan 14;5(1):e8702. doi: 10.1371/journal.pone.0008702 (PMC2806825; doi:10.1371/journal.pone.0008702)

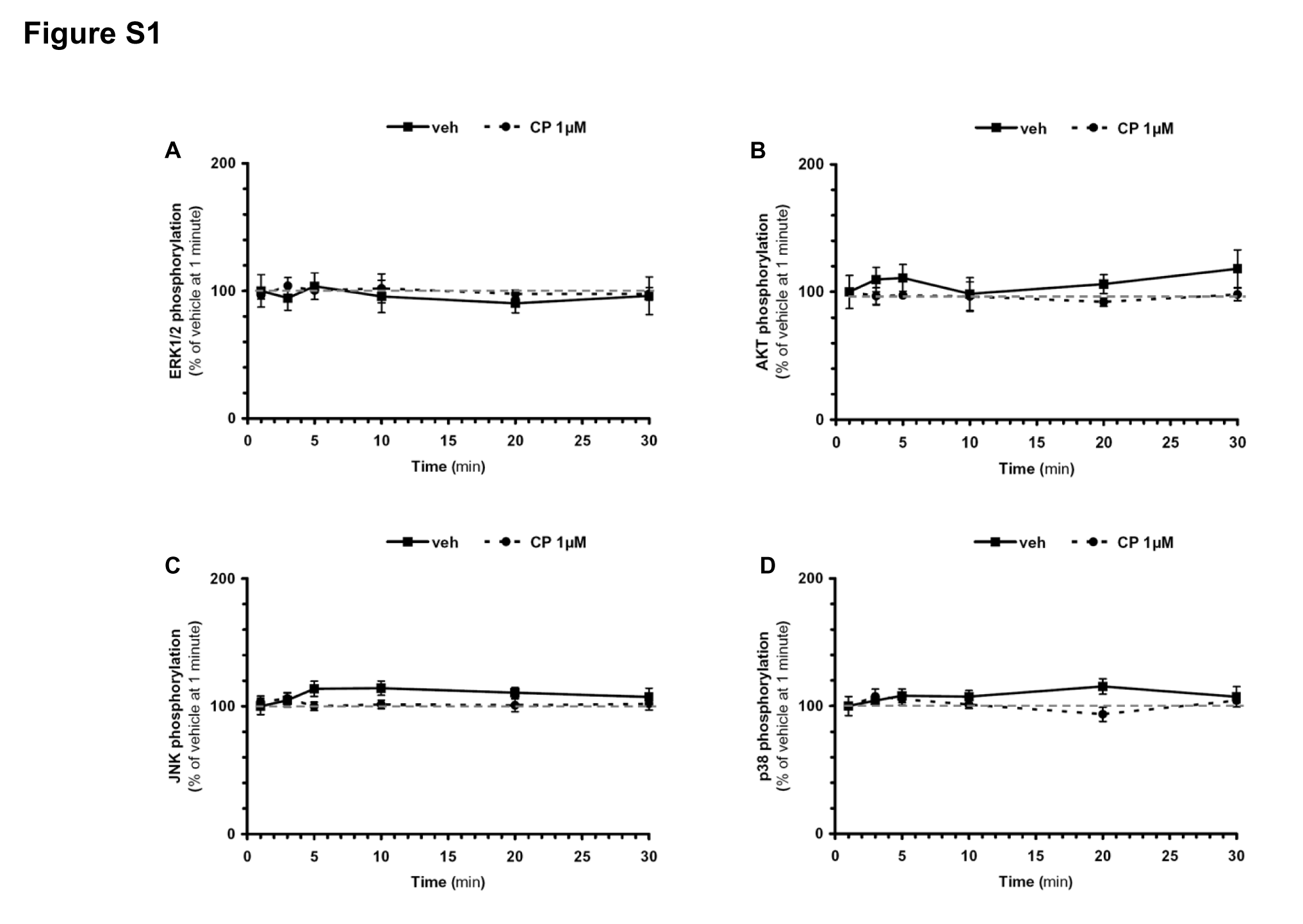

Supplement: Figure S1 — Kinase activity in wild-type. (0.45 MB TIF) [file pone.0008702.s001.tif]

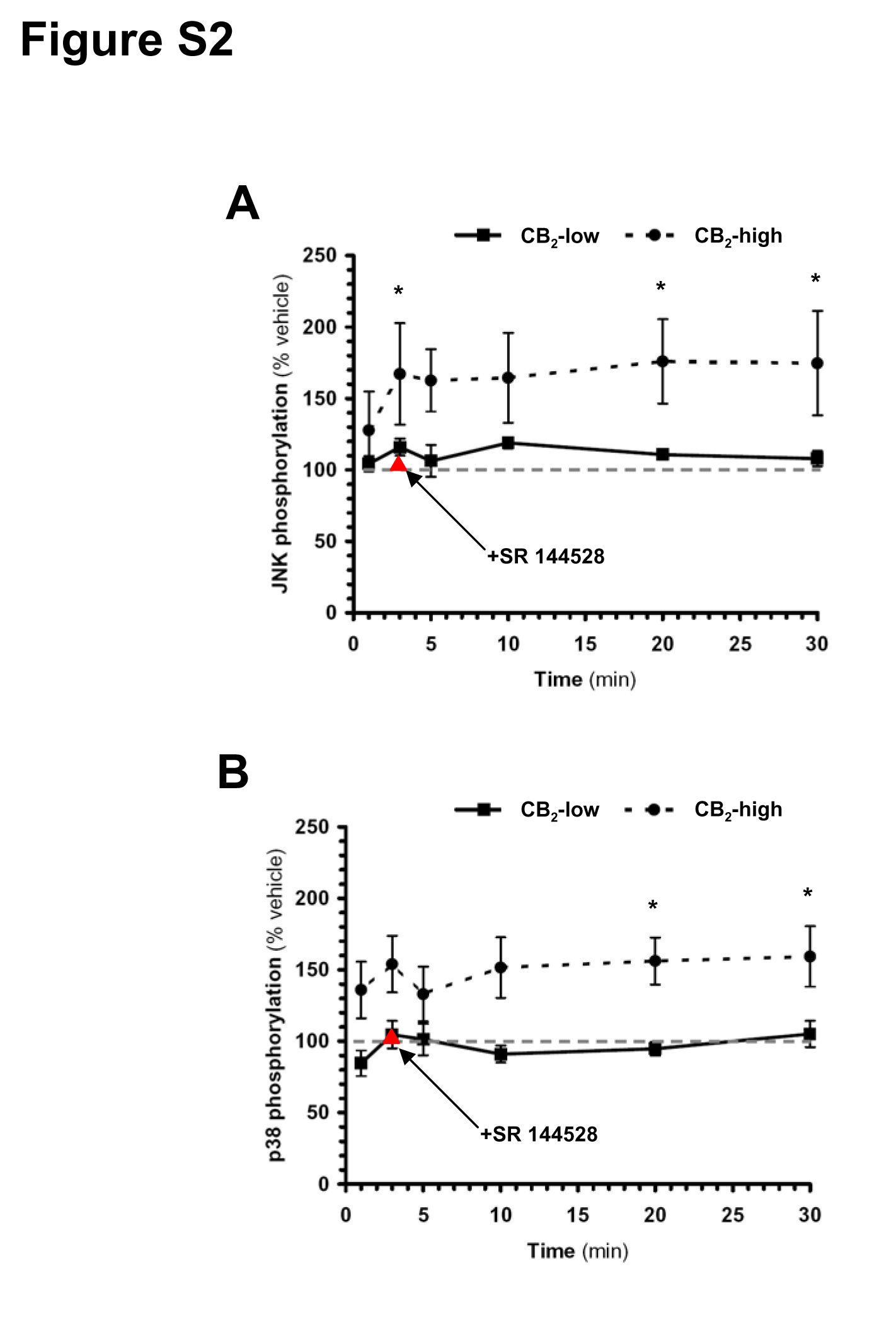

Supplement: Figure S2 — p38 and jnk activity. (0.49 MB TIF) [file pone.0008702.s002.tif]

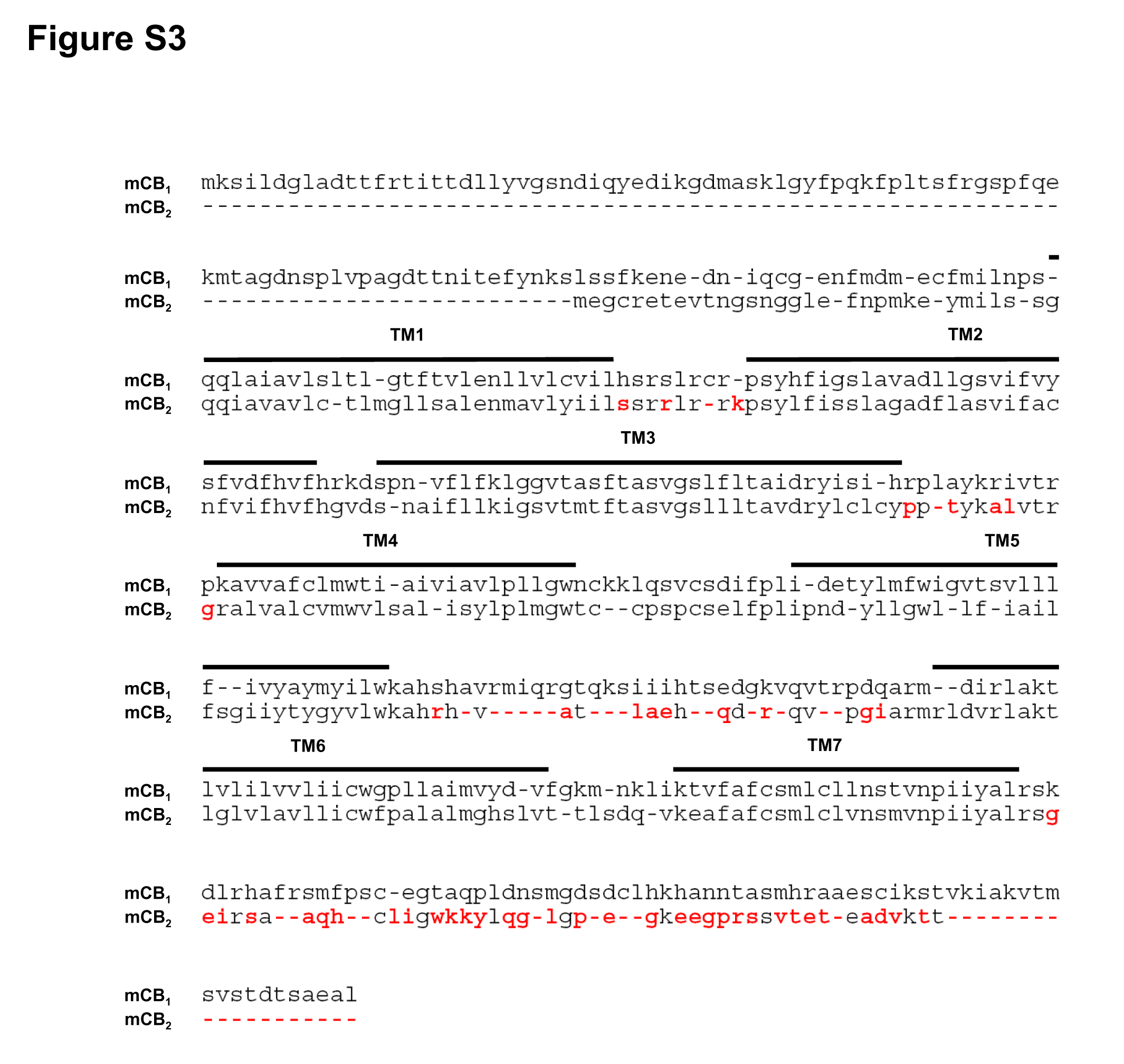

Supplement: Figure S3 — Sequence alignement between mouse CB1 and mouse CB2. (0.77 MB TIF) [file pone.0008702.s003.tif]

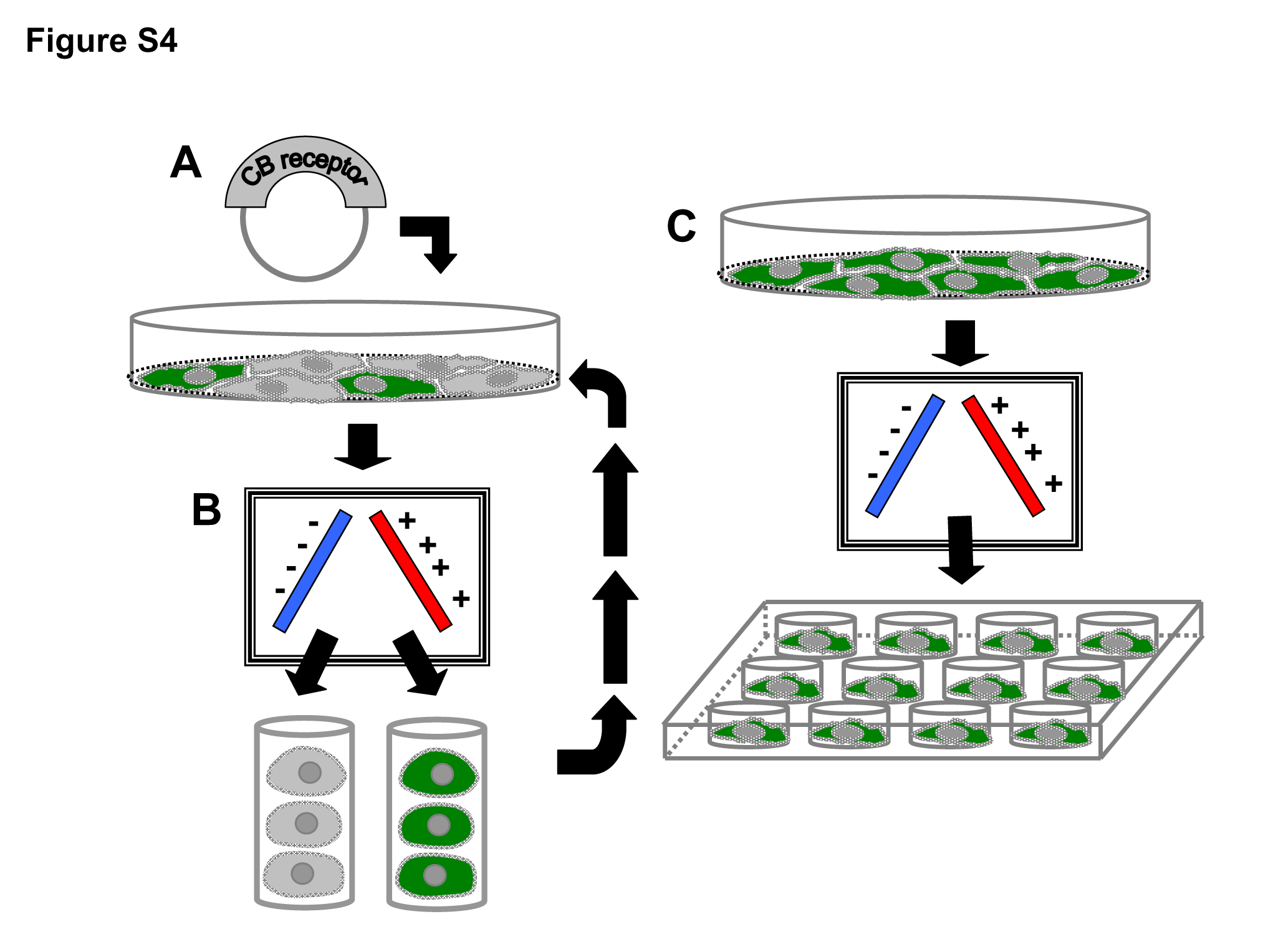

Supplement: Figure S4 — Scheme outlining the development of stable clones. (0.48 MB TIF) [file pone.0008702.s004.tif]
