# Supplementary material for: The Expression Level of CB1 and CB2 Receptors Determines Their Efficacy at Inducing Apoptosis in Astrocytomas
Source: PLoS One. 2010 Jan 14;5(1):e8702. doi: 10.1371/journal.pone.0008702 (PMC2806825; doi:10.1371/journal.pone.0008702)
Supplement: Table S1 — qPCR identification of cannabinoid receptor stable subclones. (0.04 MB DOC) [file pone.0008702.s005.doc]

**Table S1**: qPCR identification of cannabinoid receptor stable subclones

| DBT CB_1_ subclones | qPCR | | DBT CB_2_ subclones | qPCR | |
| --- | --- | --- | --- | --- | --- |
|  | **CB_1_** | **CB_2_** |  | **CB_1_** | **CB_2_** |
|  | **(cycle threshold)** | |  | **(cycle threshold)** | |
| E5 | 14.69 | no ct | 1D6 | no ct | 13.96 |
| G1 | 15.82 | no ct | 2F5 | no ct | 15.02 |
| C11 | 16.4 | no ct | 2C10 | no ct | 15.03 |
| A3 | 17.67 | no ct | 1C7 | no ct | 16.33 |
| E7 | 17.64 | no ct | 2H1 | no ct | 18.06 |
| C2 | 19.14 | no ct | 2G4 | no ct | 19.17 |
| E3 | 22.37 | no ct | 2D4 | no ct | 22.19 |
| E1 | 24.52 | no ct | 2H6 | no ct | 22.21 |
| B6 | 26.04 | no ct | 1D8 | no ct | 23.12 |
| A8 | 27.61 | no ct | 2D3 | no ct | 23.35 |
| A2 | 31.11 | no ct | 2G5 | no ct | 24.12 |
| C1 | 32.35 | no ct | 1C12 | no ct | 31.13 |
